# Enteropathogenic *Escherichia coli* O157 Strains from Brazil

**DOI:** 10.3201/eid0901.020072

**Published:** 2003-01

**Authors:** T. Eric Blank, David W. Lacher, Isabel C.A. Scaletsky, Hailang Zhong, Thomas S. Whittam, Michael S. Donnenberg

**Affiliations:** * University of Maryland School of Medicine, Baltimore, Maryland, USA; † Michigan State University, East Lansing, Michigan, USA; ‡ Universidade Federal de São Paulo, São Paulo, Brazil

**Keywords:** *Escherichia coli* infections, classification, phylogeny, base sequence, sequence alignment, fimbriae, bacterial, dispatch

## Abstract

We describe two serogroup O157 *Escherichia coli* strains from Brazilian infants with diarrhea. A variety of assays indicate that these strains belong to the enteropathogenic, not the enterohemorrhagic, pathotype. These strains possess a novel *bfpA* allele encoding the type IV pilin characteristic of typical enteropathogenic *E. coli* strains. Our results emphasize the pitfalls of classifying pathogenic *E. coli* by serogroup.

Enterohemorrhagic *Escherichia coli* (EHEC) O157:H7 is the serotype most commonly associated with hemorrhagic colitis and the hemolytic uremic syndrome (*1*). EHEC strains share with enteropathogenic *E. coli* (EPEC), a leading cause of infant diarrhea in developing countries, the ability to induce the attaching and effacing effect on host cells. This property is specified by a pathogenicity island that includes the *eae* gene encoding the outer membrane adhesin intimin. EPEC are defined by this attaching and effacing phenotype or, at a molecular level, by the presence of the *eae* gene and the absence of the genes for Shiga toxins (*2*). Typical EPEC strains have a large plasmid that encodes bundle-forming pili and the localized adherence phenotype, while atypical strains lack these properties. EHEC differ from EPEC in that they produce Shiga toxins but not bundle-forming pili.

Here we describe two strains of the O157 serogroup identified as part of an ongoing epidemiologic survey of pathogenic *E. coli* in Brazil (*3*). Strain SC373/2 was isolated from a 9-month-old infant in Joinville, Santa Catarina, in 1997, and RN587/1, from a 7-month-old infant in Natal, Rio Grande do Norte, in 1998. Both patients had acute diarrhea of 7 days’ duration, accompanied by vomiting, fever, and moderate dehydration. These strains were the only enteropathogenic bacteria isolated from the patients’ stools. Rotavirus and cryptosporidia were not detected.

We used a combination of phenotypic assays and DNA sequencing to further characterize these isolates. DNA probe testing was performed by established methods (*4*). A fragment of the *eae* gene was amplified by polymerase chain reaction (PCR) and sequenced as described (*5*). Serotyping was performed at the *E. coli* Reference Laboratory (University Park, PA). Tests of localized adherence, autoaggregation, and attaching and effacing were performed as described (*6*,*7*). The *bfpA* gene was amplified by PCR and sequenced as described (*8*). Multilocus sequence typing was performed as described (*9*).

We found a number of indications that these O157 strains are not EHEC but unexpectedly fall within the EPEC pathotype. First, both strains tested probe negative for the Shiga toxin (*stx*) genes characteristic of EHEC. Second, both strains carried an *eae* (intimin) gene, identified by sequencing as an α allele characteristic of the group of strains known as EPEC 1, rather than the γ allele characteristic of O157:H7 EHEC (C.L. Tarr and T.S. Whittam, unpub. data). As expected of both EPEC and EHEC, both strains are capable of attaching and effacing as demonstrated by using the fluorescence actin staining test. *E. coli* strains that are *eae*^+^ and *stx–* are by definition, EPEC (*2*). Third, both strains lacked the H7 flagellar antigen, although they were motile. The H antigens were difficult to type; RN587/1 reacts weakly with H8 antiserum. Fourth, both strains rapidly fermented sorbitol, a phenotype lacking in most O157:H7 strains. Fifth, the nucleotide sequences of part of the coding regions of 14 chromosomal loci encoding proteins with housekeeping functions indicated a close relationship of these strains with the EPEC 1 clonal group. The DNA sequences of the two O157 strains were identical over a total of 2,240 codons. Comparison of the multilocus sequence data to the reference strains K-12, EDL-933 (O157:H7; EHEC 1), and E2348/69 (O127:H6; EPEC 1) indicated that the EPEC O157 isolates were most closely related to E2348/69. The percentage of nucleotide difference in the total of 6,720 bp was 0.72 ± 0.10 (E2348/69), 2.66 ± 0.20 (K-12), and 2.95 ± 0.21 (EDL-933). Phylogenetic analysis with these sequences from a diverse collection of strains, including EPEC, EHEC, and other pathotypes, likewise shows that RN587/1 and SC373/2 are much more closely related to EPEC 1 than to EHEC O157 strains (Figure 1). Both strains also tested probe positive for the EPEC adherence factor plasmid, which is associated with localized adherence.

**Figure 1:**
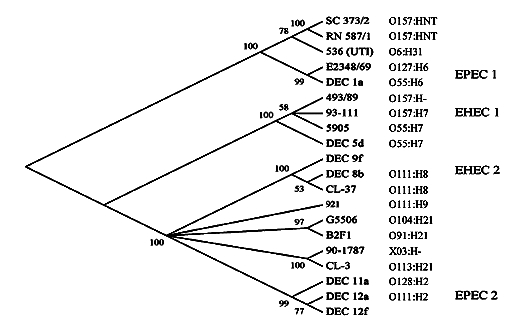
Clonal phylogeny of *Escherichia coli* strains of various pathotypes inferred from distances at synonymous sites in sequences of 13 concatenated loci (*mutS* was not included) by using the neighbor-joining algorithm. This consensus tree has numbers at each node, representing the percentage of bootstrapped trees in which the node was observed. SC373/2 and RN587/1 are the two O157 strains from Brazil. The other pathogenic strains included in the figure are described in Reid et al. (*9*). The serotype of each strain (when available) appears to the right of its designation. HNT denotes a nontypeable flagellar antigen.

Finally, both strains carried the *bfpA* gene encoding bundlin, the structural subunit of the bundle-forming pilus expressed by typical EPEC strains but not by EHEC. Both strains are also capable of autoaggregation and localized adherence, the two phenotypes associated with expression of bundle-forming pili. The *bfpA* gene isolated from these strains has a unique sequence (GenBank accession number AF474407). We call this version of *bfpA* the β6 allele. When compared to the α and β *bfpA* alleles described previously (*8*), the β6 *bfpA* allele maps between the β1 allele and a cluster of the β2–β5 alleles (Figure 2). The predicted β6 bundlin amino acid sequence maps between the β1 and β2 and the β3–5 pilin proteins.

**Figure 2:**
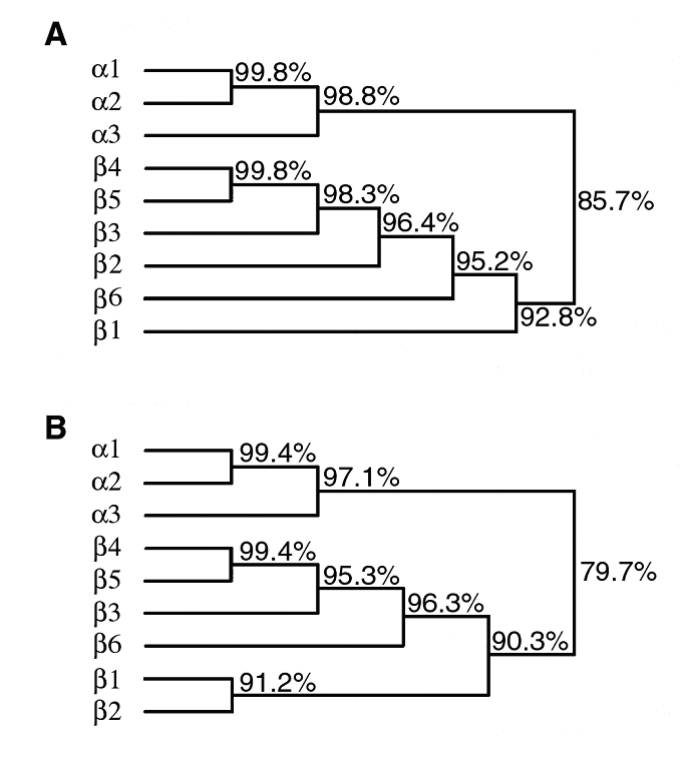
Dendrograms depicting the relationships among *bfpA* alleles (A) and predicted bundlin proteins (B). Percentage identities are indicated.

Our findings emphasize the fact that *E. coli* with the O157 O antigen are not always EHEC but may belong to other pathotypes. Scotland et al. described Shiga toxin–negative O157:H8 *E. coli* strains isolated from children with diarrhea (*10*). These strains were positive for attaching and effacing and localized adherence phenotypes and are therefore EPEC, although, unlike the strains we describe, they were negative for the adherence factor probe. EPEC strains of the O157:H45 serotype have been noted as agents of diarrhea in a large outbreak in Japan and isolated cases in Germany, Japan, and Thailand (*11*–*13*). These previously described O157 EPEC strains were not tested for bundlin alleles, and their phylogenetic relationships to other pathogenic *E. coli* have not been reported. However, the results of several studies have indicated that O157 strains lacking the H7 flagellar antigen and *stx* genes, which were not necessarily EPEC, are clonally distinct from O157:H7 strains and often from each other (*14*–*16*). Such findings suggest that recombination events causing the O antigen gene cluster to specify the O157 antigen have occurred in multiple *E. coli* lineages, including an EPEC 1 strain of an unknown serotype that is the progenitor of the strains described here. The results described in this study also highlight the pitfalls of classifying pathogenic *E. coli* by serogroup.
